# DNA methylation-mediated control of learning and memory

**DOI:** 10.1186/1756-6606-4-5

**Published:** 2011-01-19

**Authors:** Nam-Kyung Yu, Sung Hee Baek, Bong-Kiun Kaang

**Affiliations:** 1 National Creative Research Initiative Center for Memory, Department of Biological Sciences, College of Natural Sciences, Seoul National University, Seoul 151-747, Korea; 2 Creative Research Initiative Center for Chromatin Dynamics, Department of Biological Sciences, College of Natural Sciences, Seoul National University, Seoul 151-747, Korea; 3 Department of Brain and Cognitive Sciences, College of Natural Sciences, Seoul National University, Seoul 151-747, Korea

## Abstract

Animals constantly receive and respond to external or internal stimuli, and these experiences are learned and memorized in their brains. In animals, this is a crucial feature for survival, by making it possible for them to adapt their behavioral patterns to the ever-changing environment. For this learning and memory process, nerve cells in the brain undergo enormous molecular and cellular changes, not only in the input-output-related local subcellular compartments but also in the central nucleus. Interestingly, the DNA methylation pattern, which is normally stable in a terminally differentiated cell and defines the cell type identity, is emerging as an important regulatory mechanism of behavioral plasticity. The elucidation of how this covalent modification of DNA, which is known to be the most stable epigenetic mark, contributes to the complex orchestration of animal behavior is a fascinating new research area. We will overview the current understanding of the mechanism of modifying the methyl code on DNA and its impact on learning and memory.

## Cytosine methylation in mammals

In mammals, methylation at symmetric CpG dinucleotides in genomic DNA is important for heritable gene silencing and regulation of gene expression [1]. It is one of the primary epigenetic mechanisms for the regulation of gene transcription along with the various histone modifications such as methylation, acetylation, SUMOylation, ubiquitination, and phosphorylation. DNA methylation has been shown to play essential roles in genomic imprinting, X chromosome inactivation, and maintenance of genome stability [1,2].

**Figure 1:**
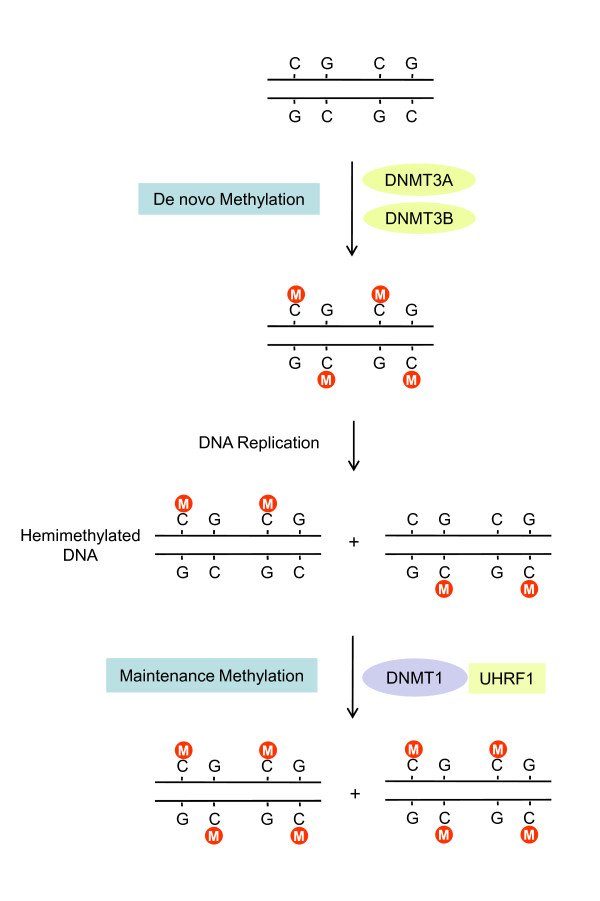
**Overview of mammalian cytosine methylation**. In mammals, cytosine methylation is necessary for regulation of DNA sequences and the following gene expression patterns. DNMT1 is active on hemimethylated DNA, which assists the maintenance of genomic methylation. The recruitment of DNMT1 to hemimethylated DNA is mediated through its interaction with UHRF1. DNMT3A and DNMT3B function as *de novo *methyltransferases, and they methylate the cytosine of previously unmethylated CpG dinucleotides on both strands.

The addition of a methyl group from SAM (*S*-adenosyl-L-methionine) substrates to the cytosine is catalyzed by DNA (cytosine-5)-methyltransferases. Three DNA methyltransferases, DNMT1, DNMT3a, and DNMT3b, have functional enzymatic activity in mammals (Figure 1) [3,4]. DNMT1 has been called a "maintenance methyltransferase" as it has a substrate preference for hemimethylated DNA over unmethylated DNA. DNMT1 interacts with the DNA replication machinery during the S phase of dividing cells so that the methylation pattern is reliably copied to the daughter strand [5]. DNMT3a and DNMT3b are regarded as "*de novo *methyltransferases" since they can methylate the cytosine of CpG dinucleotides previously unmethylated on both strands, altering the epigenetic information content. They play crucial roles in establishing genomic methylation patterns during cell differentiation [6]. However, this functional distinction is not always obvious since DNMT1 also displays *de novo *methyltransferase activity, and its substrate preference is limited in a cellular context-dependent manner [3].

The three *DNMT *genes display differential expression profiles in the central nervous system. DNMT1 is highly expressed in neurons from embryogenesis through adulthood [7,8]. DNMT3b expression is observed in neural progenitor tissue only during early embryogenesis. DNMT3a is expressed from late embryogenesis to adulthood with a peak during the early postnatal period, then its level declines but remains detectable in the post-mitotic neurons of the adult brain [9,10]. Aberrant regulation of DNMT expression has been shown to be related to drug abuse [11,12], suicide [13], and psychiatric disorders such as schizophrenia and bipolar disorder [14,15].

Given that the DNMTs exhibit little or no innate sequence specificity beyond the CpG dinucleotide [3], the manner in which these enzymes find a specific target DNA sequence in mammals could be a very fundamental question [4]. One of the candidate targeting molecules is UHRF1 (ubiquitin-like, containing PHD and RING finger domain 1), which has been shown to interact with hemimethylated DNA through its SET and RING-associated (SRA) domain [16]. UHRF1 is crucial for targeting DNMT1 to hemimethylated DNA, and permits the faithful transmission of genomic methylation patterns [16]. Notably, recent report observed an altered expression of this protein in the central nervous system by long-term memory formation[17]. Another reported mechanism is the use of other specific epigenetic marks such as histone methylation marks for binding to hemimethylated DNA. It has been shown that DNA methylation is dependent on previously shaped histone methylation marks. In addition, Polycomb group proteins link histone methylation and DNA methylation [18-20]. Small non-coding RNA-mediated guidance of DNMT is also a fascinating candidate mechanism, which has yet to be well described in mammals [4].

Whereas the enzymes for cytosine methylation are well characterized, the existence of enzymes for active demethylation is controversial [21]. Passive demethylation, which can occur without DNMT activity during cell proliferation and does not require enzyme activity, is widely accepted. In contrast, it has been doubted whether active removal of methyl residues from 5-methylcytosine would occur after cellular differentiation because the methyl mark on cytosine residues conferred by DNMT is known to be the most stable epigenetic modification due to its covalent nature. During the past decade, accumulating reports have presented observations of active demethylation [22-28]. Although there are several conceivable mechanisms for demethylation and evidence supporting them [21], these remain inconclusive. Nucleotide excision repair-based mechanism involving growth arrest and DNA damage-inducible 45 (Gadd45α or Gadd45β) proteins is considered a candidate [22,29-31]. Gadd45 proteins are known as stress-inducible non-enzymatic proteins that regulate cell cycle arrest and promote the DNA repair reaction, coupling the deamination and glycosylation, and then filling in the bare cytosine. Another candidate is the ten-eleven translocation 1 (TET1) protein, that has been shown to convert 5-methylcytosine of DNA to 5-hydroxymethylcytosine, raising the possibility that DNA demethylation may be a TET1-mediated process [32]. Further, all three TET proteins (Tet1, Tet2, and Tet3) have been reported to catalyze a similar reaction [33].

Approximately 60%-70% of CpGs in the mammalian genome are highly methylated, the exception being the CpG-dense area in the vicinity of a promoter, which is called the CpG island and displays generally low, but tissue-specific methylation levels [4]. Given that cytosine methylation in the promoter represses the transcription of the gene, there are two modes of repression: (1) methyl cytosine can repel transcriptional activators, or (2) attract transcriptional repressors that have methyl cytosine-binding domains and recruit proteins such as histone deacetylase, which facilitate the formation of the silent chromatin state [34,35]. These methyl cytosine-binding proteins include methyl CpG-binding domains (MBDs) and methyl CpG-binding protein 2(MeCP2). In humans, the MeCP2 mutation is the well-known cause of autism spectrum disorder, Rett Syndrome [36], indicating the importance of proper interpretation of methyl cytosine marks by MeCP2.

## Cytosine methylation patterns undergo dynamic change in the central nervous system

It was a long-held belief that cytosine methylation patterns would be cell-type specific and stable. The only opportunity to change the methylation pattern was thought to be during the cell division period when DNA is newly synthesized with bare cytosine residues. Unexpectedly, even though postmitotic neurons are terminally differentiated and no longer proliferate, DNA methyltransferases were found to be abundantly expressed in neurons [7], and their enzymatic activity was also significant in the brain [8]. These findings raised the possibility that DNA methylation patterns might be dynamically changing in the brain for some unknown roles. Accumulating evidence has shown that cytosine methylation could be altered in postmitotic neurons by neural activity [25,26,37] or during behavioral change in response to external signals [23,24,38].

A well-known example of this is cytosine methylation pattern formation by early life experience. Maternal care is known to affect the stress response in adulthood in rats. Weaver et al. showed that this enduring effect is mediated by a cytosine methylation change on specific gene loci [23,39]. High levels of maternal licking and grooming and arched-back nursing (High-LG) demethylate the promoter of the glucocorticoid receptor (GR) gene exon 1_7 _in the hippocampus, and this change persists into adulthood [23]. This change can be reversed by treating the brain with L-methionine, so that the GR promoter is highly methylated, causing the low GR expression levels and high stress response as seen in the offspring of low-LG mothers [40]. This demonstrates that methylation change plays a key role in behavioral control through regulating GR expression. In addition, methylation of the glutamic acid decarboxylase 1 (GAD1) promoter, which is negatively correlated with mRNA levels, is decreased in the offspring of high-LG mothers [41]. Early life stress such as maltreatment or repeated maternal separation, which causes defective behavior in adulthood, also changes the methylation level of brain-derived neurotrophic factor (BDNF) or arginine vasopressin (AVP) [42,43]. In humans, higher methylation levels at the glucocorticoid receptor gene (Nr3c1) exon 1_7 _promoter was found in the brains of suicide victims having a history of childhood abuse compared to those without such a history [44]. These findings show that DNA methylation patterns are shaped by experiences during the critical period of postnatal brain development, which persists throughout the lifespan.

An increasing number of reports have shown the dynamic nature of cytosine methylation in the brain. Social avoidance behavior that was induced by chronic social defeat stress given in adulthood coincided with demethylation of the stress hormone corticotrophin-releasing factor (*Crf*) gene and increase of its mRNA in the mouse hypothalamus [45]. There are studies showing that DNA methylation levels change according to age [46-48]. Electroconvulsive treatment into the hippocampal dentate gyrus area, which induces neuronal activity by direct electrical current injection in vivo, decreases the methylation level of specific regulatory regions of BDNF and fibroblast growth factor-1 (FGF) genes, correlatively increasing their mRNA and protein expression levels [22]. In addition, remarkable works done by JD Sweatt and his colleagues showed that contextual fear conditioning also induces methylation change in neural plasticity-related genes such as BDNF, reelin, PP1, and calcineurin [24,38,49].

In a rodent primary neuron culture, high potassium-induced neuronal depolarization demethylates the BDNF exon IV promoter (according to the new nomenclature) [50], correlating with a corresponding increase in mRNA after neural activity [25,26]. MeCP2 basally represses BDNF expression, and is phosphorylated and dissociated from the BDNF promoter in response to neural activity, as the promoter is demethylated. Other repressing chromatin complexes are also detached, whereas phospho-CREB, the transcriptional activator, binds to the promoter. Picrotoxin-induced chronic network activity also causes methylation changes in specific loci of the BDNF and reelin promoter, which in turn alters the miniature excitatory postsynaptic current (mEPSC) frequency [37].

Methylation levels in the BDNF gene region are dynamically changed in postmitotic neurons both in vitro and in vivo as has been reported in several studies from different laboratories. The rodent BDNF gene has nine exons, the individual mRNAs of which are differentially regulated according to the tissue and brain region [50]. Epigenetic mechanisms have been reported to control the transcription of the BDNF gene, and DNA methylation seems to participate in the regulation. However, according to different reports, the specific promoters undergoing changes in methylation are quite diverse. Chronic network activity caused by picrotoxin treatment of cultured neurons [37] or contextual exposure to the living animals demethylates the promoter of exon I [38]. The methylation level of this exon I promoter was also shown to be correlated with object recognition memory task performance [51]. The exon IV promoter is demethylated in the hippocampus by high potassium treatment that induces membrane depolarization [25,26], as well by contextual fear-conditioning given to animals [38]. The promoter is methylated in the prefrontal cortex by maltreatment in the early postnatal period [42], but is not affected by electroconvulsive treatment (ECT) to the dentate gyrus of the hippocampus [22]. ECT demethylates the promoter of the coding exon IX [22], which is methylated by early life maltreatment [42]. Collectively, it seems that the regulatory elements of the BDNF gene are differentially methylated or demethylated according to age or brain region, and the types of upstream signals.

As stated above, many studies have shown that external stimuli can alter the DNA methylation levels of behaviorally important genes in the brain. Mostly, the DNA methylation level is negatively correlated with mRNA or protein level. DNMT inhibitors can reverse the increased methylation and decreased transcription, which also blocks behavioral plasticity, suggesting the biological importance of these changes. Therefore, we would like to describe the contribution of methyl change to learning and memory in the next part.

## DNA methylation contributes to synaptic or behavioral long-term plasticity

Alteration of neuronal gene expression pattern is required for long-term memory formation or for synaptic plasticity which is thought to underlie learning. Since DNA methylation is involved in neural activity-induced transcriptional changes, methylation might be important in the process of in vivo long-term memory formation. This key hypothesis was first examined by JD Sweatt and his colleagues. They showed by a series of elegant experiments that DNMT activity is required for associative memory formation and induction of long-term potentiation (LTP) [24,52]. The DNMT inhibitor 5-azadeoxycytidine (5-AZA), or zebularin infusion into the hippocampus immediately after contextual fear conditioning reverses the methylation and downregulation of PP1, a memory suppressor gene, impairing the formation of a fear memory [24]. Interestingly, methylation levels in the hippocampus return to baseline in 24 hrs, although the fear memory is maintained and is still dependent on the hippocampus at that time point [24]. This indicates that in the hippocampus, DNA methylation might not be a mechanism of contextual fear memory maintenance, but is a regulatory mechanism of transient gene expression. However, we cannot exclude the possibility that the methylation state of other genes might be persistently changed in hippocampal neurons during the time the memory is dependent on the hippocampus. Not only fear conditioning but also other types of learning, such as cocaine-induced conditioned place preference memory, were recently shown to require DNA methylation in the hippocampus [53].

Since DNA methyltransferase inhibitor drugs have a non-specificity problem [54], genetic manipulation of DNMT provides another valuable strategy for understanding the causal relationship. Genetic approaches targeting DNMTs have been performed to assess their importance in synaptic function or learning and memory.

Conventional DNMT1, or DNMT3a and DNMT3b deletion causes genomic hypomethylation and embryonic lethality [6,55], indicating that proper DNA methylation is required for normal development, but make it impossible to study the function of this modification in adulthood using these mice. DNMT1 ablation in neuronal precursor cells eventually causes global DNA hypomethylation, cell death during early postnatal development, and neonatal lethality [56]. Conditional DNMT3a knockout in neuronal precursor cells leads to a specific methylation pattern change, neuromuscular function abnormalities, and premature death [57]. These findings suggest that an appropriate DNA methylation pattern is required for postnatal neuronal survival or function, but cannot be used to evaluate the contribution of DNA methylation to adult brain function.

To assess the role of DNA methylation in the mature brain, DNMT1 has been deleted specifically in the precursors of postnatal excitatory neurons in the dorsal forebrain in Emx1, a promoter-driven conditional mutant mouse line, which survived into adulthood but showed abnormal development of somatosensory barrel cortex and impaired thalamocortical long-term potentiation [58]. When DNMT1 is deleted under the control of the calmodulin-kinase IIα (CaMKIIα) promoter [56], neither the DNA methylation level of endogenous retroviral repeats nor neuronal survival is affected. Interestingly, double knockout of DNMT1 and DNMT3a, mediated by the CaMKIIα-cre system results in smaller cell sizes, impaired hippocampal LTP, enhanced LTD, and deficits in spatial and contextual fear memory formation, whereas the single knockout lines of each gene display no abnormalities [59]. Immune function-related genes are upregulated and the global or specific DNA methylation levels are decreased in the double knockout mouse forebrain. In particular, Stat1, which is involved in neural plasticity and the interferon pathway, is upregulated at the mRNA level and decreases at the methylation level in NeuN-positive neurons. These results suggest that DNA methylation is important for synaptic plasticity and learning, probably through affecting the expression of plasticity-related genes. Another implication is that DNMT1 and DNMT3a play complementary roles in postmitotic excitatory neurons, although they have distinct enzymatic properties. Starting from this interesting finding, numerous questions arise. The detailed molecular mechanisms of how their roles are redundant and how their deficiency causes demethylation of specific sequences are elusive. Since the absence of DNMT was prolonged in the abovementioned study, the behavioral effect and the gene expression pattern appearing in the microarray might reflect only chronic influence and not the inducible role of DNMTs during learning.

Although some genes, including BDNF, are demethylated after learning, with a corresponding increase in mRNA, no study has shown if DNA demethylation is necessary for memory consolidation. This is because the demethylation mechanism remains unclear. A recent interesting report described how activity-induced gene Gadd45b is required for activity-dependent upregulation of BDNF [22]. The role of Gadd45b for DNA demethylation might be challenged in the learning and memory paradigm in the future.

## Role of CpG methylation for maintaining long-lasting memory

Persistence is one of the most enigmatic features of memory. The prevailing hypothesis is that memory is encoded by the altered synaptic strength in the complex neuronal circuits; however, the detailed mechanism remains elusive. The DNA modification hypothesis of memory storage was first proposed by J.S. Griffith and H.R. Mahler in 1969 [60]. Since molecular turnover is a naturally continual process, DNA might be the one storage molecule that could maintain the learned information for the lifetime. In 1984, F. Crick [61] postulated that the maintenance molecule to overcome the dissipation of acquired changes would form multimers or at least dimers with each monomer having modified (+) or unmodified (-) modes. Even if one (+) component is exchanged by a newly synthesized (-) molecule by natural turnover, the hypothetic maintenance enzyme will quickly convert it to the modified (+) mode. In this way, once the (+)(+) conformation is acquired, the (+)(+) conformation would be maintained, which matches the feature of DNMT1. In 1999, R. Holliday indicated the cytosine methylation state of specific gene loci as a candidate crucial mechanism of memory storage [62].

It was only recently that a first report by J.D. Sweatt and his colleagues appeared demonstrating that DNA methylation is required for the maintenance of memory [49]. Contextual fear memory formation and its initial maintenance depend on the hippocampus, but it is generally believed that memory undergoes systems consolidation over approximately 3 weeks, so that the remote memory becomes dependent on the prefrontal cortex, including the anterior cingulate cortex (ACC), and independent of the hippocampus [63,64]. To test whether memory maintenance requires DNA methylation, Miller et al. [49] looked at the ACC region rather than the hippocampus. After contextual fear conditioning, hypermethylation of the calcineurin (CaN) gene was maintained for at least 30 days. A correlative decrease of calcineurin mRNA and protein also persisted for at least a month. When a DNMT inhibitor was injected into the ACC 29 days after training, DNA methylation on CaN decreased and 30-day memory was impaired. These findings suggest that the DNA methylation and demethylation processes are ongoing in the ACC region and that this dynamic balance is required for memory maintenance. It would be worth testing whether the same mechanism is applicable to other types of long-lasting memories such as conditioned taste aversion. Similarly, retrieval of conditioned place preference memory was recently reported to depend on DNA methylation in the prelimbic cortex [53].

**Figure 2:**
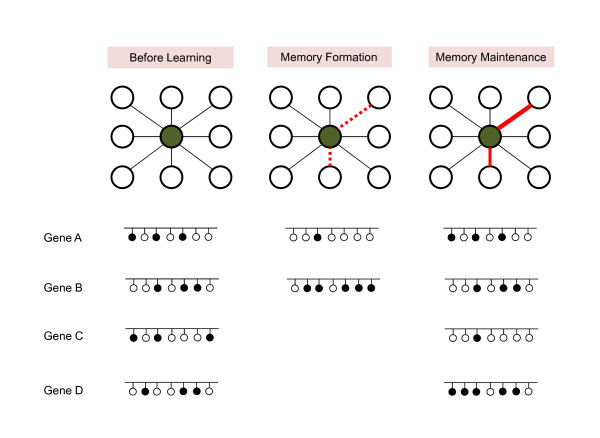
**DNA methylation in learning and memory**. Upper: One neuron (central green circle) has a number of connections with other neurons (peripheral circles). Lower: Each small circle represents a CpG site in a regulatory element of the gene. Filled circles indicate methylated CpGs and the white circles unmethylated CpGs. When the animal has a new experience ("Memory formation" state), some connections are activated (red dashed lines). Transient waves of gene upregulation or downregulation are required for memory formation and could be mediated by temporal modifications of DNA methylation. Memory suppressor genes (Gene B), such as PP1, are transcriptionally downregulated through DNA methylation, and plasticity-inducing genes, such as BDNF or reelin (Gene A), are upregulated with DNA demethylation. The methylation states of these genes are restored to the baseline level after memory consolidation. When the memory has been stabilized ("Memory maintenance" state), the neurons exhibit an altered profile of connection strength (compared to "Before learning" state in upper panel). We expect that the gene expression patterns in neurons need to be different from those before learning in order to maintain this modified combination of connection strengths. Maintaining this altered gene expression pattern might involve a stable change in DNA methylation. Calcineurin is a known example for Gene D that is increased in cytosine methylation and decreased in mRNA level. However, Gene C with decreased methylation and increased mRNA level that is associated with learning and memory has not been discovered in adult animals.

One might ask how the modification of DNA in the nucleus that has a cell-wide effect could be involved in maintaining a specific memory [65]. A neuron has thousands of synapses connecting to a number of other neurons, and there is a high possibility that it participates in multiple memories through different synapses. It seems quite certain that modification of DNA in the nucleus itself cannot differentially affect each synapse without synapse-specific changes. Therefore, we believe that DNA methylation in itself is insufficient to store the memory. However, as recent evidence suggests, it is likely that maintaining the DNA methylation pattern by balanced methylation-demethylation activity is required for memory maintenance [49]. After a learning experience, the profile of synaptic strength or property in a participating neuron would be changed (Figure 2). To maintain this altered pattern of connections, neurons would need to contain certain amounts of their gene products, which could be stably controlled at the transcriptional level by CpG methylation at the regulatory element. If an imbalance is induced by DNMT inhibitor, the neuron would lose its capacity to maintain the strength of connections, impairing the memory storage [49]. If we are correct in our hypothesis that a neuron participates in multiple memories and that DNA methylation is required to maintain the entire synaptic properties of a neuron, DNMT inhibitor injection into a specific brain region would affect the different types of memory stored in that region. In addition, this persistent change should be subtle, such that the element could still be responsive to the upcoming signals to encode another memory. Conversely, there might be a limit to the number of memories a single neuron can participate in.

## Conclusions and perspectives

Contrary to the traditional view, an ever-growing number of reports indicate that cytosine methylation in neurons seems to change quite dynamically after birth. Recent studies have shown that cytosine methylation is important for both memory formation and memory maintenance (Figure 2). In addition to its critical involvement in the transcriptional regulation of genes controlling memory consolidation processes, cytosine methylation has also been shown to be required for retaining the memory. However, the detailed mechanism is hardly known and many questions remain. What might be the upstream pathway of temporal regulation of DNMTs or the demethylation machinery? What other genes and how many genes in the genome will be affected during a behavioral learning process? Which mechanism would confer target specificity? There is skepticism that experience-mediated DNA methylation changes in neurons are too small to be of biological importance, although it is statistically significant. Clear answer to the questions about mechanism will help to evaluate whether the observed methylation changes in the brain are indeed biologically significant.

Moreover, most studies on the role of DNA methylation in the brain have analyzed the DNA methylation state using whole tissue lysates by methods such as bisulfite sequencing, methylation-specific PCR (MSP), or methyl CpG-specific immunoprecipitation. Due to the high heterogeneity of cell types in the brain, it is difficult to determine the type of cells in which the methylation level is changed and the number of cells that undergo the changes. In addition, the extent of methylation change could be blurred by the mixed population of cells. This problem might be solved by utilizing a recently reported nucleus analysis protocol [45,66]. Cell type-specific nuclei are labeled by transgenic expression of a fluorescence protein or by immunostaining. Using flow cytometry, only the target nuclei are separated from the whole tissue-purified nuclei, and these can then be analyzed in terms of DNA methylation. This method might be utilized to determine whether the detected changes after learning occur in neurons or astrocytes, or in other specific subpopulation of neurons. Interesting results are expected from labeling the activated neurons using reporter proteins driven by an immediate early gene promoter [67,68]. Analysis of the methylation pattern in this specific set of neurons would provide valuable information regarding the activity-induced regulation of cytosine methylation.

Another potentially useful means of detecting DNA methylation state at the cell level is by using the MSP-ISH technique [69]. Although to date there have been an insufficient number of studies using this method, possibly due to the methodological difficulties, the concept of observing the methylation pattern at the cell level could be used in the future to examine how many cells have the changes, and to analyze other factors such as neuronal structure concomitantly with methylation. It might be also combined with the various tracing methods used to examine neuronal connections.

Furthermore, it seems that cytosine methylation in neurons would be modulated delicately and dynamically for behavioral plasticity, which is distinct from the conspicuous cytosine methylation change in developmental or disease states. Conceivably, extensive change in DNA methylation patterns could cause abnormal or pathological states for the neuron; therefore, there might be a mechanism of neuron-specific tight regulation of DNA methylation.

Since DNA methylation is related to long-term behavioral alteration, it might be a good therapeutic target for treating long-term behavioral disorders. L-Methionine treatment in adulthoods has been shown to reverse the behavioral effect of lack of maternal care [40]. The content of diet, such as folate or vitamin C, is closely related to cytosine methylation levels [70,71]. DNMT inhibitors, such as 5-AZA, which are currently used for cancer treatment, might be utilized as treatment for behavioral disorders.

## Competing interests

The authors declare that they have no competing interests.

## Authors' contributions

NKY, SHB and BKK conceived of the review and drafted the manuscript. All authors read and approved the final manuscript.
